# Iodine Status of Women and Infants in Russia: A Systematic Review

**DOI:** 10.3390/ijerph17228346

**Published:** 2020-11-11

**Authors:** Rimma Korobitsyna, Andrey Aksenov, Tatiana Sorokina, Anna Trofimova, Nikita Sobolev, Andrej M Grjibovski, Valery Chashchin, Yngvar Thomassen

**Affiliations:** 1 Arctic Biomonitoring Laboratory, Northern (Arctic) Federal University Named After M. V. Lomonosov, Naberezhnaya Severnoy Dvini 17, 163002 Arkhangelsk, Russia; a.s.aksenov@narfu.ru (A.A.); t.sorokina@narfu.ru (T.S.); a.trofimova@narfu.ru (A.T.); n.sobolev@narfu.ru (N.S.); Yngvar.Thomassen@stami.no (Y.T.); 2 Central Scientific Research Laboratory, Northern State Medical University of the Ministry of Healthcare of the Russian Federation, Troitskiy Ave. 51, 163000 Arkhangelsk, Russia; andrej.grjibovski@gmail.com; 3 Department of Health Policy and Management, Al-Farabi Kazakh National University, Almay 050040, Kazakhstan; 4 Department of Epidemiology and Modern Vaccination Technologies, Sechenov First Moscow State Medical University (Sechenov University), 119991 Moscow, Russia; 5 West Kazakhstan Marat Ospanov Medical University, Aktobe 0300190, Kazakhstan; 6 North-Western State Medical University named after I.I. Mechnikov, Kirochnaya ul. 41, 191015 Saint-Petersburg, Russia; valerych05@mail.ru; 7 Institute of Ecology, National Research University Higher School of Economics, Myasnitskaya str. 20, 101000 Moscow, Russia; 8 National Institute of Occupational Health, P.O. Box 5330 Majorstua, N-0304 Oslo, Norway

**Keywords:** iodine status, median UIC, pregnant women, women of reproductive age, infants

## Abstract

This systematic review presents a critical synthesis of the available information on the iodine status among women and infants in Russia. Literature search was performed in accordance with PRISMA guidelines using PubMed, Scopus Web of Science databases as well as eLIBRARY—the Russian national source. Altogether, 277 papers were identified and 19 of them were eligible for the review. The data on median urinary iodine concentration (UIC) in women and infants from 25 Russian regions were presented. A substantial variability in UIC across the country with no clear geographical pattern was observed. Despite substantial heterogeneity in research methodology and data presentation the results suggest that the iodine status among pregnant women and infants in Russia is below the recommended levels. Our findings demonstrate that iodine deficiency is a re-emerging public health problem in Russia. Urgent public health measures on national, regional and individual levels are warranted.

## 1. Introduction

Iodine (I) is an essential element required for synthesis of the thyroid hormones triiodothyroine (T3) and thyroxine (T4) which participate in regulating multiple metabolic processes. The main symptoms of severe I deficiency (ID), termed I deficiency disorders (IDDs), include endemic goiter, hypothyroidism, cretinism, decreased fertility rate, increased infant mortality, and mental retardation. ID is described as the single greatest global cause of preventable mental impairments [1]. More recent studies have found that even mild ID is associated with lower educational levels of children and cognitive impairment. Thyroid hormones are essential for brain development and this is especially true in early pregnancy prior to the onset of fetal thyroid function [2,3]. Iodine deficiency (ID) is recognized by the World Health Organization (WHO) as the most common cause of damage brain [4].

Pregnant and lactating women need adequate intake of I for maternal T4 production which is of special importance for fetal development in the first trimester and for brain development during the first years of life [1]. Increased renal clearance during pregnancy increases I requirements for pregnant women [5]. According to the World Health Organization (WHO), the recommended nutritional I intakes are 150 µg for adolescents (above 12 years) and adults; 250 µg for pregnant and lactating women, respectively [6,7]. About 90% of I absorbed dose eventually appears in the urine. Therefore, the urinary I content is considered as a good marker for the recent dietary intake of I. Although I excretion vary considerably both between and within days, these variations tend to even out on a population level [8]. The WHO epidemiological criteria for assessing I status based on median urinary I concentrations (UIC) (µg/L of I) for school-age children (>6 years) is as follows; <20 (severe ID); 20–49 (moderate ID); 50–99 (mild ID) and 100–199 (adequate intake). For pregnant women the criteria are <150 (insufficient) and 150–249 (adequate intake).

ID is a major health challenge worldwide. Although in 1990 the World Health Assembly and the World Summit for Children established a global goal to eliminate severe IDD by 2000 it is obvious that the goal was not reached but some progress in improving global I status has been achieved [9]. In the former Soviet Union severe IDD was reported eliminated by the 1960s and Government programs directed at IDD prevention were discontinued in the 1970s [10]. After break-up of the USSR in 1991 IDD re-emerged in nearly all former Soviet republics including the Russian Federation where the population is facing insufficient I intake [11]. Russia is the largest country in the world by area and has a multiethnic population. Thus, national data may mask regional variations in both I intake and prevalence of IDD.

Cold environments require additional amount of thyroid hormones [12], enhancing triiodothyronine (T3) production from thyroxine (T4) to activate the heat production at local level in brown adipose tissue which is essential in the cold adaptation of Arctic residents [13]. Hypothyroidism increases human susceptibility to cold-induced health effects [14]. Due to extreme climatic conditions in a large part of Russia studies on the prevalence of IDD and adequate I supplementation are important public health issues in Russia. However, the evidence on the prevalence of ID in Russia published in the international peer-reviewed literature is scarce. At the same time, research data published in local biomedical journals is of limited availability to the international audience and may suffer from methodological limitations [15]. 

Thus, the main purpose of this systematic review is to critically summarize the evidence from international and Russian peer-reviewed literature on the I status among the most vulnerable to IDD population groups, namely, women of reproductive age and infants in order to identify gaps in knowledge to be filled for development and implementation of effective IDD prevention programs in Russia.

## 2. Materials and Methods 

This study is a systematic review designed in accordance with the PRISMA Extension for Scoping Reviews (PRISMA-ScR) [16,17]. A systematic literature search was carried out in PubMed, Scopus and Web of Science databases as well as eLIBRARY [18]—the national Russian source of scientific literature. Search terms and combinations of terms in PubMed, Scopus, Web of Science and eLIBRARY included: «(iodine or iodine deficiency or iodine status) and (pregnancy or pregnant women) and (fertile women or women of reproductive age) and/or (newborn or infants) and (urine or median urinary iodine concentration) and (Russia)». Duplicate publications were checked and eliminated.

**Figure 1:**
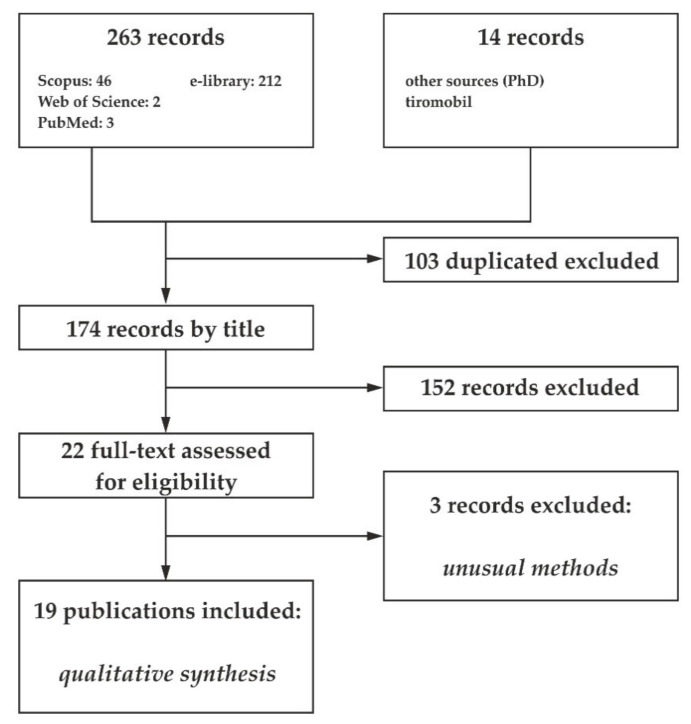
Flow chart of articles selection procedure.

Initially, 277 articles were identified (Figure 1). After removing duplicates as a result of a search in electronic databases and viewing links to articles, 174 (103 were excluded) publications remained. Application of inclusion/exclusion criteria and careful examination of the abstracts resulted in exclusion of 152 studies. Of the remaining 22 papers 3 were excluded for the following reasons: 1—no full text available, 2—questionable methods for determining I status. The remaining 19 studies were eligible for qualitative synthesis.

The following inclusion/exclusion criteria were used to identify all possible peer-reviewed journal articles in a consistent, reliable, and objective manner with exclusion of those ineligible for the study:

Inclusion criteria:

- Study subjects were residents of the Russian Federation;
- Studies were conducted in Russia from 1991 to 2019;
- Women of reproductive age including non-pregnant, pregnant and lactating women, newborns and infants;
- WHO standardized guidelines for I status are were used; and
- Median UIC data was available.

Exclusion criteria:

- Animal studies;
- Insufficient data on sample collection/preparation/analysis;
- Review articles;
- Patients with diagnosed thyroid disease and/or other chronic diseases; and
- Patients treated with radioactive iodine isotopes viz. ^123^I, ^124^I, ^125^I, and ^131^I.

Evaluation of the quality of peer-reviewed articles. 

Two researchers (one Ph.D. and one Master student) worked independently of each other using the PRISMA flow chart. Assessment of individual studies for systematic errors using the Cochrane Collaboration Risk of Bias tool [17] was performed. Disagreements were resolved by consensus with the third author (MD). After quality assessment, 19 articles remained eligible for the qualitative synthesis.

## 3. Results and Discussion

The characteristics of the study participants, region of residence, methods for determining I status, as well as median UIC in the 19 studies are presented in Table 1.

Although only 19 original studies were included in the review many of them included results of observations in several groups and several regions. Including pregnant, non-pregnant, lactating women, as well as children in one study was common. Moreover, in a few studies data on UIC were presented for pregnant women both with and without I supplementation. Thus, the results below are grouped by categories. 

### 3.1. Non-Pregnant Women of Reproductive Age

Three of the 19 reviewed papers included data on non-pregnant women with the range of UIC from 47 μg/L to 127 μg/L. Two of the three suggest moderate [31] or mild [37] I deficiency. 

### 3.2. Pregnant Women 

Median UIC values for pregnant women were reported in 16 articles covering 43 individual studies. In 9 of the studies women received IS. Thus, the results for pregnant women are stratified by IS status. 

### 3.3. Pregnant Women without I Supplementation

Fifteen of the 19 reviewed papers included data on pregnant women aged 18–45 years with median UIC values in pregnant women ranging from 33 to 192 μg/L [21,27]. This indicates a serious cause for concern, as many of these values are below the WHO recommendations for median UIC in pregnant women.

If we consider the change in the concentration of median UIC by trimester, there was no universal pattern, but in general, the following can be noted:

- A decrease in median UIC throughout pregnancy reaching a minimum in the III trimester was described in—4 studies (Rostov, Cheboksary, Smolensk) [22];
- a decrease in median UIC in the second trimester with an increase in the third trimester. Median UIC values in the third trimester are almost equal to the values in the first trimester (Anapa, Ivanovo), or even exceed by more than 20% (Nizhnekamsk) [22]; and
- an increase in median UIC in the second trimester compared with the first, and then a return to the concentration to the values of the first trimester (Novocheboksary, Almetyevsk, Kirov) [22,23].

This decrease in median UIC is most likely due to that women of reproductive age may not have sufficient I intake to maintain thyroid health and metabolism with potential adverse effects on the neurological health of the developing fetus.

### 3.4. Pregnant Women with I Supplementation

Nine of the 19 reviewed papers included data on pregnant women aged 18–42 years taking IS with median UIC values between 27 and 260 μg/L [20,30]. As reported, only in Nizhny Novgorod, when taking IS 300 μg potassium iodine per day, the required concentration of median UIC for pregnant women is achieved. Due to IS, the median UIC value increased slightly in the Moscow, Smolensk, Penza, Saratov, Astrakhan, Krasnodar, and Tomsk regions. Which is also indicates an ID in these regions within the pregnant women, since these median UIC levels are below the WHO recommended range.

The change in the median UIC by trimester in women taking IS, demonstrate a pattern in decreasing the median UIC throughout pregnancy reaching a minimum of 27 μg/L (Astrakhan) [30], which corresponds to a moderate severity of I deficiency.

### 3.5. Nursing Women and Infants

Three of the 19 reviewed papers included data for mother–infant couples. The range of median UIC values for these pairs was 20–155 μg/L [36] and 25–190 μg/L [36] for mothers and their children, respectively. Among these the following features can be noted:

- About half of the studies showed similar median UIC values in mother and child (for example, Sakha Republic,69 and 67 μg/L [36]; Khabarovsk 55 and 69 μg /L [37], respectively);
- the other studies indicate that median UICs in newborns exceeded those measured in mothers by 2–3.5 times (e.g., in Amurs where the median UIC values in mother and child are 20 and 75 μg/L [36], respectively);
- of interest are the median UICs in mother-newborn pairs in the Vanino village where the values are 31 and 96 μg/L, respectively. For mothers, this median UIC level is much lower than that recommended and corresponds to a moderate severity of ID, while in newborns the median UIC is close to the recommended value;
- the lowest median UICs were observed in the Jewish Autonomous Region (Birobidzhan); 27 and 25 μg/L, for mother and infants, respectively [36]; and
- the highest median UICs were reported from the Kamchatka territory (Petropavlovsk-Kamchatsky) reaching 155 and 190 μg/L, respectively, for mothers and children [36].

In all studies, with the exception of the Kamchatka territory, the recommended median UIC level for lactating women was not achieved. Children also fail to achieve the optimal level of median UIC, with the exception of the Kamchatka territory and the Khabarovsk territory (Vanino village)

Two of the 19 reviewed papers included data of lactating women taking IS. The range of median UIC values in nursing women who took IS was 41–118 μg/L [20]. However, the median UIC values were below the WHO recommended level for lactating women.

Iodine intake is an important determinant of I status, which is difficult to assess. Therefore, median UIC is one of the most appropriate and commonly used indicator of I status. Reference intervals of the I concentrations recommended by WHO [38] help researchers to characterize the I status of the population in a proper way using median UIC as a marker of I status. The limitation of spot urine sampling as the matrix for I status determination is the inability to evaluate the individual I status due to a significant within-day and day-to-day variability of the individual’s I intake [39]. Because of that, all the studies reviewed in the present manuscript used median UIC as an indicator of population’s I status.

It should be noted that most of the reviewed articles do not provide sufficient description of the analytical method used in measurement of I in urine. Most of the measurements were performed by the cerium-arsenate reaction or the colorimetric Sandell–Kolthoff-methods which were introduced decades ago [40]. These methods with some modifications are still used and even recommended by WHO for epidemiological studies where I status is to be assessed [38]. However, more accurate methods based on inductively coupled plasma mass spectrometry (ICP-MS) have been introduced few decades ago and are today a gold standard for UIC measurements [41]. The Sandell–Kolthoff-method with some modification is still quite often used when relatively cheap method is needed. A recent study has shown [39] that a microplates Sandell–Kolthoff-method obtained similar results as ICP-MS confirming that the Sandell–Kolthoff-method is a reliable alternative method for UIC measurements.

Quality assurance information in the reviewed articles, however, are unfortunately not available for documentation of detection limits, accuracy and repeatability. Thus, there is no complete confidence that the reviewed data of the UICs of the Russian population are accurate.

The median UICs measured in pregnant women across Russia clearly indicate that almost all groups studied had not sufficient I intake. An exception is the group of pregnant women from the Pacific coastal area (Petropavlovsk-Kamchatski) presumably due to high consumption of lean white sea fish and other sea products (the yearly fish consumption per capita in the Pacific region is of 31.5 kg in contrast to the national average of 21.7 kg) [42].

In countries neighboring Russia (Norway, Denmark, Mongolia, China, Belarus, Ukraine) iodized salt is a source of I [43,44,45,46,47,48,49]. In Norway and Denmark, products such as milk, dairy products, fish and fish products make up almost 80% of the dietary I intake [43,44]. In Ukraine, along with iodized salt, one of the most accessible sources of I is algae [46]. In Belarus, the mandatory use of high-quality iodized salt in the food industry has led to the elimination of I deficiency among the population [48].

The experiences of neighboring countries and the USSR show that salt iodization is an effective strategy to prevent ID [50]. In the United States, the median UIC for women of reproductive age is also below the WHO recommended level [51]. China generally had adequate I intake with significantly higher average UIC among pregnant women in Shandong (244 μg/L) than in Tianjin (159 μg/L). No difference was found in median UIC during pregnancy in Shandong. The I status of pregnant women in Tianjin and Shandong was sufficient, but various changes in median UIC and thyroid function during pregnancy were reported. The authors of the study call for attention to I nutrition of pregnant women, even in areas with sufficient I content [52]. 

In Norway, a study of pregnant women and newborns showed that low I intake (lower than ~150 μg/day) was associated with fetal growth retardation in three exposure indicators: I from food, median UIC, and use of I supplements. In addition, low dietary I intake (lower than ~100 μg/day) and lack of I supplementation has been associated with an increased risk of preeclampsia. The use of I supplements can satisfy the increased need for I during pregnancy. The risk of hypothyroidism is reduced in women with severe I deficiency, while studies in women with mild to moderate deficiency are not consistent [53]. This inconsistency in findings is related to a range of measurement, design and location factors [2,4]. However, an increasing amount of evidence suggests that even mild ID is associated with mild cognitive difficulties particularly in expressive language and working memory tasks [2,3,4,54]. Starting I supplementation in the first trimester can lead to temporary “thyroid stunning”, which can adversely affect the developing fetus. Therefore, it is necessary to pay special attention to the intake of I by women before pregnancy, as well as during pregnancy [53].

The three studies raise concerns regarding excessive and uncontrolled excess intake of I, which can lead to deleterious health effects for women and children.

When interpreting the quantitative data on I status assessment in epidemiological studies of the prevalence, distribution, and severity of ID it is important to pay special attention to a number of environmental confounders and effect modifiers such as cold climate, vitamin D deficiency and some chemical food contaminants that may influence the I-related health effects. 

For example, the vitamin D deficiency might be a contributing risk factor to non-autoimmune hypothyroidism which is also associated with ID [55]. This is specifically important for the Russian population residing in northern areas due to lack of solar UV-radiation and low consumption of seafood which is one of the main nutritional sources of I and vitamin D. Thus, vitamin D deficiency in Arctic areas may enhance the impact of ID on the vulnerable groups of the population such as pregnant women, infants, and children. 

Other life-style challenges in Russia today are nutritional habits resulting in increased obesity [56], food may also be an important source of environmental contaminants such as persistent organic pollutants (POPs), lead and mercury. These environmental contaminants have similar adverse effects as ID on the neurocognitive development of children among other impacts, such as the perturbation of thyroid hormones [57]. Thus, any risk assessment of exposure to these environmental neuro-toxicants should take into consideration the potential confounding of the I status. 

Iron (Fe) and zinc deficiencies continue to be global health problems and especially iron deficiency anemia (IDA) during pregnancy and infancy. IDA is a strong factor for cognitive, motor and socioemotional impaired development of children [58]. Recent studies have shown that among individuals with IDA the thyroid hormone metabolism is impaired most likely because of the reduced activity of the Fe-dependent enzyme—thyroid peroxidase. This argue for improving the Fe-status in areas of overlapping deficiencies, not only to combat IDA but also ID with dual-supplementation with both Fe and I [59,60].

In a recent study there were found multidirectional associations of serum concentrations of POPs and I containing thyroid hormones [61]. Researchers found that perfluorooctanesulfonic acid (PFOS) was positively associated with TSH (thyroxine-binding globulin); polychlorinated biphenyls (PCBs), hexachlorobenzene (HCB), and nonachlors were inversely associated with T3, T4; and new emerging compounds viz perfluorodecanoic acid (PFDA) and perfluoroundecanoic acid (PFUnDA) were also inversely associated with T3.

Effects of persistent contaminants were found on vitamin metabolism, immune functioning and hormones in the arctic wildlife as well [62].

The Russian guidelines recommend a daily I intake of at least 150 μg for women of reproductive age, whereas a daily dose of 250 μg is recommended for residents of endemic ID areas. Despite these recommendations, only 9% of Russian women take dietary vitamin and mineral supplementation [63]. It has been reported that three quarters of pregnant women are affected by micronutrient deficiency [63]. All women and children need I deficiency prevention. Special attention should be paid to women of reproductive age before they become pregnant, because of the time lag to fully synthesize I into thyroid hormones.

The main reason for the lack of significant progress in Russia to prevent ID is the absence of a national-wide regulatory act on prevention of IDD and a centralized system for monitoring the implementation of preventive measures [11]. Only in 2020, the use of iodized salt has become mandatory in Russia when catering children in schools and institutions of secondary education [64]. At present, a draft Federal Law “On Prevention of Iodine Deficiency Disorders” dated 27 March 2019 has been developed, which will be important for establishing a further legal foundations of state policy [65].

## 4. Conclusions

A substantial variability in UIC among Russian women and infants across the country with no clear geographical pattern was observed during the studied period. Despite substantial heterogeneity in research methodology and data presentation the results suggest that the iodine status among pregnant women and infants in Russia is below the recommended by the WHO levels. Our findings demonstrate that ID is a re-emerging public health problem in Russia. Urgent public health measures on national, regional and individual levels are warranted. Further studies are required for assessing the I status in the population of Russia.

## Figures and Tables

**Table 1:** Iodine status in Russian pregnant women and newborns from studies that met inclusion criteria.

Area of Residence	Data		Subjects	Median UIC (μg/L)	Analytical Method	References
	Year of Publication	Year of Sample Collection				
***Moscow region***						
*	2011		75 pregnant women on I trimester (aged 18–42 years) (using nutritional supplements)	128	Cerium-arsenite reaction	[19]
*	2010		Initially 75 pregnant women	128	Cerium-arsenite reaction	[20]
			Group 1: 59 pregnant women receiving IS ^a^ 200 μg/day as KI	124		
			Group 2: 16 pregnant women receiving IS ^a^ 300 μg/day as KI	196		
			Nursing mothers from Group 1	118		
			Nursing mothers from Group 2	82		
			16 infants of mothers from Group 1	180		
			7 infants of mothers from Group 2	200		
***Ivanovo region***						
*Ivanovo city*		2005	84 pregnant women	93		[21]
*Ivanovo city*	2008	2001–2003	84 pregnant women aged 26 ± 4 years		Colorimetric	[22]
			14 I trimester	116		
			25 II trimester	82		
			45 III trimester	94		
***Smolensk region***						
*Smolensk city*	2008	2004	150 pregnant women aged 25 ± 5 years		Colorimetric	[22]
			50 I trimester	93		
			50 II trimester	86		
			50 III trimester	51		
*	2011		119 pregnant women on I trimester (aged 18–42 years)	63	Cerium-arsenite reaction	[19]
			(taken nutritional supplements)			
*	2010		Initially 119 pregnant women	63	Cerium-arsenite reaction	[20]
			Group 1: 50 pregnant women receiving IS ^a^ 200 μg/day as KI	84		
			Group 2: 69 pregnant women receiving IS ^a^ 300 μg/day as KI	121		
			Nursing mothers from Group 1	41		
			Nursing mothers from Group 2	70		
			22 infants of mothers from Group 1	174		
			19 infants of mothers from Group 2	136		
***Tatarstan republic***						
	2008	2003	180 pregnant women (aged 25 ± 5 years)		Colorimetric	[22]
*Al’met’evsk*			92 pregnant women:	112		
*(54.9 °N, 52.17 °E)*			13 I trimester	102		
			26 II trimester	129		
			53 III trimester	110		
*Nizhnekamsk*			88 pregnant women:	164		
*(55.63 °N, 51.82 °E)*			21 I trimester	155		
			36 II trimester	148		
			31 III trimester	192		
*		2005	182 pregnant women	150		[21]
***Kirov region***						
*Kirov city*	2005	2004	92 pregnant women (aged 26 ± 4 years)		Colorimetric	[22,23]
			25 I trimester	69		
			32 II trimester	87		
			35 III trimester	73		
*		2004	92 pregnant women	73		[21]
***Nizhny Novgorod region***						
*	2011		220 pregnant women on I trimester (aged 18–42 years)	141	Cerium-arsenite reaction	[19]
*	2010		Initially 220 pregnant women	141	Cerium-arsenite reaction	[20]
			Group 1: 111 pregnant women receiving IS ^a^ 200 μg/day as KI	97		
			Group 2: 109 pregnant women receiving IS ^a^ 300 μg/day as KI	260		
			Nursing mothers from Group 1	77		
			Nursing mothers from Group 2	107		
			99 infants of mothers from Group 1	110		
			93 infants of mothers from Group 2	150		
***Chuvash republic***						
*Cheboksary*		2007	98 pregnant women	83		[21]
*(56.12 °N, 47.23 °E)*						
*Novocheboksarsk*						
*(56.12 °N, 47.49 °E)*						
*Cheboksary*	2005	2003	96 pregnant women (aged 26 ± 6 years)	83	Colorimetric	[22,23]
			62 pregnant women	83		
			12 I trimester	180		
			23 II trimester	94		
			27 III trimester	78		
*Novocheboksarsk*			34 pregnant women	77		
			13 I trimester	76		
			10 II trimester	84		
			11 III trimester	68		
***Bashkortostan republic***						
*Ufa city*	2004	2000–2003	Non-pregnant	127	Cerium-arsenite reaction ^b^	[24]
			Pregnant women receiving IS ^a^			
			I trimester	143		
			II trimester	120		
***Saratov region***						
*Engel’s*	2010		Group 1: 62 pregnant women	115	Cerium-arsenite reaction	[25]
*(51.46 °N, 46.12 °E)*			Group 2: 54 pregnant women receiving IS ^a^	177		
*Engel’s*	2010		Group 1: a random of 106 pregnant women	116	Cerium-arsenite reaction	[26]
			Group 2: 90 pregnant women receiving IS ^a^	164		
*	2003		123 pregnant women did not receive IS ^a^	33	Cerium-arsenite reaction ^c^	[27]
			120 pregnant women who received IS ^a^	134		
***St. Petersburg***						
*St. Petersburg city*	2017	2013-2015	184 pregnant women (aged 18-45 years)	112	Cerium-arsenite reaction ^d^	[28]
*Tyumen region*						
*	2015	1999	Pregnant women	93	Cerium-arsenite reaction	[29]
		2009	Pregnant women	124		
***Kabardino-Balkar republic***						
*Nalchik city*		2007	Pregnant women	67		[21]
***Krasnodar territory***						
*Slavyansk-on-Kuban’*	2005	2003	120 pregnant women (aged 26 ± 6 years)		Colorimetric	[22,23]
*(45.26 °N, 38.12 °E)*			17 I trimester	98		
*Anapa*			46 II trimester	91		
*(44.89 °N, 37.32 °E)*			57 III trimester	95		
*sta. Kanevskaya*						
*(46.05 °N, 38.95 °E)*						
*		2005	121 pregnant women	90		[21]
***Astrakhan region***						
*Astrakhan city*	2010	2006-2009	68 pregnant women with IS ^a^	176	Cerium-arsenite reaction ^e^	[30]
			infants	95		
			I trimester	63		
			II trimester	50		
			III trimester	27		
			67 pregnant women in the III trimester without IS ^a^	49		
			infants	39		
***Adygea republic***						
*Majkop*		2005	60 pregnant women	84		[21]
*(44.36 °N, 40.60 °E)*						
*Majkop*	2005	2005	60 pregnant women (aged 26 ± 5 years	84	Colorimetric	[22]
			32 pregnant women with IS ^a^	115		
			28 pregnant women without IS ^a^	73		
***Rostov region***						
*		2005	303 pregnant women	95		[21]
*	2005	2004	299 pregnant women (aged 25 ± 5 years)	95	Colorimetric	[22]
			55 I trimester	104		
			107 II trimester	99		
			137 III trimester	95		
***Novosibirsk region***						
*Novosibirsk city*		1994–1995	200 men and women (aged 25–34 years)	47	Cerium-arsenite reaction	[31]
***Tomsk region***						
*	2007	2003-2005	238 pregnant women (aged years 27) of whom received IS ^a^		Potentiometric ^f^	[32]
			I trimester	65		
			II trimester	96		
			III trimester	70		
			30 pregnant women of the control group III trimester without IS ^a^	60		
***Irkutsk region***						
*Irkutsk city*	2009		Pregnant women	48		[33]
			Lactating women	75		
			Lactating women with thyroid goiter	50		
*		2002	150 pregnant women	60	Cerium-arsenite reaction	[34]
***Zabaykalsky*** ***territory***						
*	2011		Pregnant women	128		[35]
***Khabarovsk territory***						
*Khabarovsk city*	2010		Mother (25)-	55	Cerium-arsenite reaction ^g^	[36]
*(48.48 °N, 135.07 °E)*			infant (25) pairs	69		
*Komsomol’sk-on-*			Mother (30)–	26		
*Amur*			infant (31) pairs	64		
*(50.55 °N, 137 °E)*			Mother (19)-	20		
*Amursk*			infant (17) pairs	75		
*(50.22 °N, 136.9 °E)*			Mother (21)-	75		
*Nikolaevsk-on-Amur*			infant (21) pairs	86		
*(53.15 °N, 140.73 °E)*			Mother (19)-	31		
*s. Vanino*			infant pairs (20)	96		
*(49.08 °N, 140.27 °E)*						
*Khabarovsk city*	2008		30 healthy women in the early postpartum period	55	Cerium-arsenite reaction ^g^	[37]
			and 22 non-pregnant women	69		
***Jewish Autonomous region***						
*Birobidzhan city*	2010		Mother (20)-	27	Cerium-arsenite reaction ^g^	[36]
			infant (22) pairs	25		
***Sakha (Yakutiya) republic***						
*Neryungri*	2010		Mother (23)-	69	Cerium-arsenite reaction ^g^	[36]
*(56.66 °N, 124.73 °E)*			infant (23) pairs	67		
***Sakhalin region***						
*Oha*	2010		Mother (26)-	49	Cerium-arsenite reaction ^g^	[36]
*(53.58 °N, 142.93 °E)*			infant (24) pairs	61		
***Kamchatka territory***						
*Petropavlovsk-Kamchatsky city*	2010		Mother (20)–	155	Cerium-arsenite reaction ^g^	[36]
			infant (23) pairs	190		

* Location was not specified. ^a^ I supplementation. ^b^ Method by Wawschinek O. et al., 1985, WHOb, 1985, Dunn J. et al., 1993 [24]. ^c^ Evaluated spectrophotometrically from the results of the Saundell–Kolthoff reaction. ^d^ On an “ImmunoMini NJ 2300” analyzer (Japan). ^e^ The method recommended by International Council for control for I deficiency disorder (IDD) (WHO, 1993), was evaluated spectrophotometrically from the results of the Saundell–Kolthoff reaction in the clinical biochemistry laboratory. The working range of determination is 20–400 μg/L. ^f^ Method using an ion-selective electrode. This method has a state certificate of metrological certification of analysis methods No. 08-47/134, 2002. ^g^ The method recommended by the International Council for control for iodine deficiency disorders (WHO, 1993), modified by J.T. Dunn (1993) [36].
